# Effect of platinum electrode materials and electrolysis processes on the preparation of acidic electrolyzed oxidizing water and slightly acidic electrolyzed water†

**DOI:** 10.1039/c8ra08929a

**Published:** 2019-01-22

**Authors:** Xiang Song, Hui Zhao, Keneng Fang, Yongshan Lou, Zongkui Liu, Chifeng Liu, Zhandong Ren, Xiaorong Zhou, Hua Fang, Yuchan Zhu

**Affiliations:** School of Chemical and Environmental Engineering, Wuhan Polytechnic University Wuhan 430023 P. R. China renzhandong@163.com zhuyuchan@163.com

## Abstract

Electrolyzed oxidizing water (EOW) can be divided into acidic electrolyzed oxidizing water (AEOW) and slightly acidic electrolyzed water (SAEW). AEOW has the characteristics of low pH (pH < 2.7) and high oxidation–reduction potential (ORP > 1100 mV). SAEW is slightly acidic (pH = 5–6) and has an ORP of 700–900 mV. AEOW and SAEW both have a certain amount of active chlorine content (ACC), so they have the characteristics of broad spectrum, rapidity and high efficiency of sterilization. At present, there is little systematic research on AEOW and SAEW preparation. However, it is very important to study the preparation process, including electrode material and electrolytic process. First, the effects of Pt electrodes with different thermal decomposition temperatures on AEOW's pH, ORP and ACC values were investigated in detail. Next, for the SAEW preparation, the process is based on the preparation of AEOW by ion-exchange membrane electrolysis, reasonably mixing the electrolyzed cathode and anode solution. The effects of technological conditions such as electrolysis time, current density and electrolyte concentration have been systematically studied, and it is expected to get SAEW with a pH value slightly less than 7, a higher ORP value and a certain amount of ACC.

## Abstract 2

The effects of platinum electrode materials and electrolysis processes on the preparation of AEOW and SAEW have been systematically studied.

## Graphical abstract

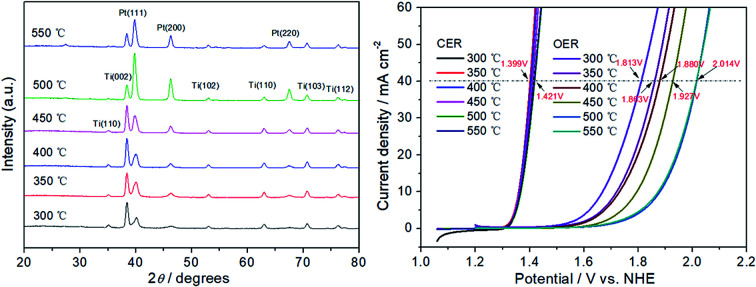

## Introduction

According to the characteristics of electrolyzed oxidizing water (EOW), it can be divided into acidic electrolyzed oxidizing water (AEOW) and slightly acidic electrolyzed water (SAEW). AEOW has the characteristics of low pH (pH < 2.7) and high oxidation–reduction potential (ORP > 1100 mV). SAEW is slightly acidic (pH = 5–6) and its ORP value is 700–1000 mV. EOW has a certain amount of active chlorine content (ACC), so it has the characteristic of broad spectrum, rapidity and high efficiency of sterilization.^1,2^ As the pH value of SAEW is close to neutral, it can reduce the corrosion of metals and the pollution of chlorine gas in the preparation process, thus possessing more extensive application potential.

EOW can seriously destroy the cytoplasm, cell membrane and cell wall of microbial cells, and increase the permeability of cell membrane, resulting in leakage of K^+^, Mg^2+^, DNA and protein. EOW can also affect the relative activity of TTC-dehydrogenase, protein synthesis activity and adenosine triphosphate synthesis pathway, thus effectively killing pathogenic microorganisms.^3–5^ EOW can kill many kinds of bacteria,^6–17^ and its killing efficiency is better than that of common food fungicides (sodium hypochlorite, ozone, chlorine dioxide, *etc.*). In addition, fungi, blood viruses and toxins can also be effectively sterilized by EOW.^18–22^ EOW is a safe disinfectant that has been confirmed harmless to the human body in various kinds of acute toxicity and subacute toxicity experiments. With the deepening of EOW research, its application scope has been expanded to medical and health care, food safety, crop growth, pest control and so on. Especially in the food industry, many research results have shown that it has good sterilization and preservation effects on fresh vegetables,^23–28^ fruits,^29–32^ fish,^33–36^ meat,^37–40^ eggs^41^ and rice.^42^

At present, there is little systematic research on EOW preparation. In 2003–2005,^43,44^ Hsu first discussed the effects of flow rate, salt concentration and temperature on AEOW preparation. Kiura^45^ found that ACC was positively correlated with NaCl concentration or electrolysis time. Moreover, it is very important to improve the efficiency and stability of the electrode. Otherwise, the energy consumption will be too high and the produced electrolyzed water will not be qualified to kill bacteria efficiently. Ru^46,47^ and Ir^48,49^ electrodes have high electrolysis efficiency but poor stability. Pt electrode has good stability,^50^ but its electrolysis efficiency of conventional electroplating platinum is not high. Therefore, from the point of view of electrolysis efficiency, the effects of Pt electrodes with different thermal decomposition temperatures on AEOW's pH, ORP and ACC values were systematically investigated in this paper. Pt electrodes were characterized and analyzed by SEM and XRD, and their electrochemical activities were analyzed by using cyclic voltammetry and linear voltammetry scanning. As for the preparation of SAEW, the reported method is to use HCl + NaCl electrolyte to carry out the electrolysis in the cell without ion-exchange membrane.^51–56^ However, this method use HCl as electrolyte, which will inevitably cause a certain degree of pollution to the environment and bring a certain risk to production. Another way to prepare SAEW is to modify the ion exchange membrane electrolysis process by reasonably mixing the electrolytic cathode solution (alkaline electrolyzed reduced water, AERW) and anode solution (AEOW) according to a certain proportion. Such preparation method can avoid the use of hydrochloric acid, but there are few studies on process optimization except Machado.^57^ Therefore, in this paper, the effects of process conditions such as electrolysis time, current density and electrolyte concentration have been systematically studied to get SAEW with a pH slightly less than 7, a higher ORP value and a certain amount of ACC.

## Experimental

### Materials

Hydrochloroplatinic acid (H_2_PtCl_6_), acetone (CH_3_COCH_3_), oxalic acid (H_2_C_2_O_4_), sodium carbonate (Na_2_CO_3_), ethanol (C_2_H_5_OH), *n*-butyl alcohol (C_4_H_9_OH) sodium chloride (NaCl), sodium thiosulfate (Na_2_S_2_O_3_), potassium iodide (KI), starch (C_12_H_22_O_11_) and sulfuric acid (H_2_SO_4_) were purchased from Sinopharm Chemical Reagent Co., Ltd., Shanghai, China.

### Electrode preparation

A Ti plate was utilized as the electrode substrate, which was sandblasted, degreased in acetone and then in boiling 10% oxalic acid at 96 °C for 1 h to produce a gray surface with uniform roughness. The Pt/Ti electrode was prepared by thermal decomposition. The precursors H_2_PtCl_6_ was dissolved in 1 : 1 volume ratio *n*-butyl alcohol and ethanol mixed solutions, in which the total metal concentration was kept at around 0.2 mol L^−1^. The process of preparation is an impregnation-pulling method. The pretreated Ti substrate was impregnated in the precursor solution for 20 s, and then slowly removed from the precursor solution at 1 mm s^−1^. A uniform liquid film was formed on the surface of the pretreated Ti substrate under the effect of viscosity and gravity. Next, the electrode was dried to evaporate the solvent at 130 °C for 10 min, and then placed in a muffle furnace at 300–550 °C for 10 min. After cooling to room temperature, the electrode was coated again. This procedure was repeated 10 times. The thermal oxidation time was 1 h for the final cycle. Platinum electrodes prepared at calcination temperatures of 300, 350, 400, 450, 500 and 550 °C are designated as Pt-300, Pt-350, Pt-400, Pt-450, Pt-500 and Pt-550, respectively.

### Electrochemical measurements

Cyclic voltammetry (CV) measurements were performed from 0 to 1.3 V in 0.5 mol L^−1^ H_2_SO_4_ solution at the scan rate of 50 mV s^−1^. After obtaining a stable cycle, the CER polarization curves were obtained by sweeping the potential from 1.00 to 1.50 V (*vs.* RHE) at a scan rate of 5 mV s^−1^ in 4.0 M NaCl (pH = 1.0). The OER polarization curves were obtained by sweeping the potential from 1.20 to 2.10 V (*vs.* RHE) at a scan rate of 5 mV s^−1^ in 0.5 mol L^−1^ H_2_SO_4_ solution. Moreover, the capacitance of H-adsorption can be used to estimate the electrochemically active surface areas (ESA) of Pt catalysts, according to the following equation (ESA = *Q*_H_/2.1 × 10^−4^ cm^2^). *Q*_H_ represents the charge exchanged during the H-adsorption and 2.1 × 10^−4^ C cm^−2^ is taken as the charge required to form a monolayer of hydrogen on a smooth polycrystalline Pt electrode.

### EOW preparation and analysis

Acid Electrolyzed Oxidizing Water (AEOW), Alkaline Electrolytic Reduction Water (AERW) and Slightly Acid Electrolyzed Oxidizing Water (SAEW) were prepared by a two-chamber ion-exchange membrane electrolyzer. The volume of cathode and anode chamber was 50 mL, respectively. The anode is Pt/Ti and the cathode is Ti, which the electrode area is 1 cm^2^. In the process of electrolysis, add some NaCl (0.01–0.09 wt%) as electrolyte, control of current density in 30–80 mA cm^−2^, electrode spacing is 2 cm and electrolysis time is 10–90 min. After the electrolysis, the AEOW was obtained in the anode region, and the AERW was obtained in the cathode region. SAEW was obtained by mixing cathode solution (AERW) and anode solution (AEOW) with volume ratio 1 : 1. The pH of the mixture was measured by a pH meter, the ORP value was measured using an ORP apparatus, and the ACC was measured by spectrophotometric method using the TMB (3,3,5,5-tetramethyl-benzidine) colorimetric method. In this method, TMB was oxidized by the ACC to form a yellow product and its concentration was analyzed immediately using a spectrophotometer (UV-2102PC, UNICO, US) at 450 nm.

## Results and discussion

**Fig. 1:**
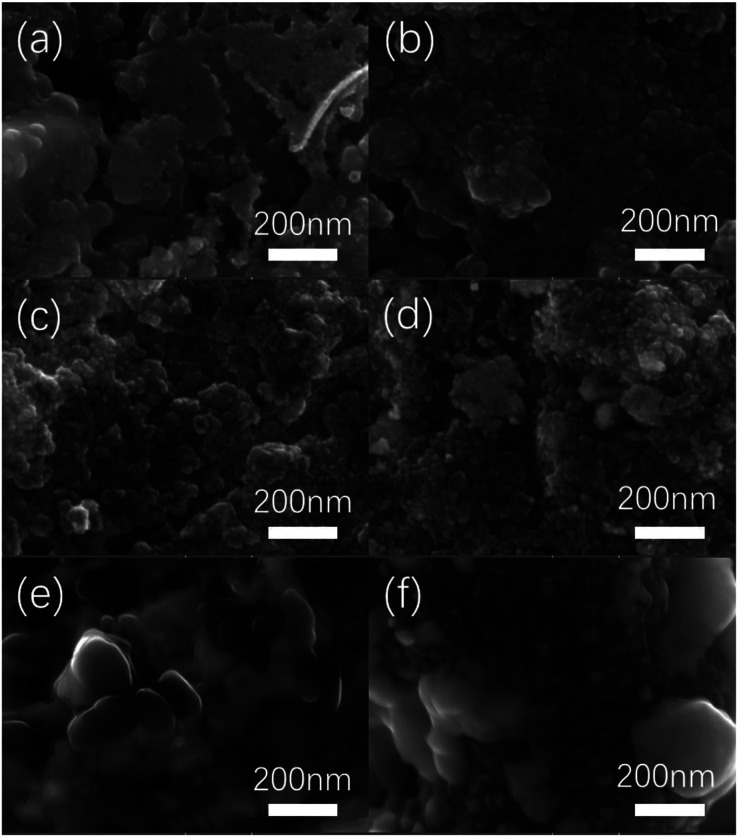
SEM diagram of Pt electrodes with different thermal decomposition temperatures. ((a) 300 °C, (b) 350 °C, (c) 400 °C, (d) 450 °C, (e) 500 °C, (f) 550 °C).

Fig. 1 is a SEM diagram of platinum electrodes at different thermal decomposition temperatures. When the thermal decomposition temperature is 300 °C, the morphology of the electrode is relatively smooth without no obvious particles. When the temperature of electrode preparation is raised to 350 °C, some tiny particles can be seen in the surface of electrode with the size 30–40 nm. When the preparation temperature continues to rise to 400–450 °C, some agglomeration appears on the electrode surface. Such agglomeration is caused by high temperature sintering. With the increase of roasting temperature, agglomeration phenomenon becomes more and more obvious. When the decomposition temperature is increased to 500–550 °C, many particles with a diameter of 100–200 nm has appeared on the electrode surface.

**Fig. 2:**
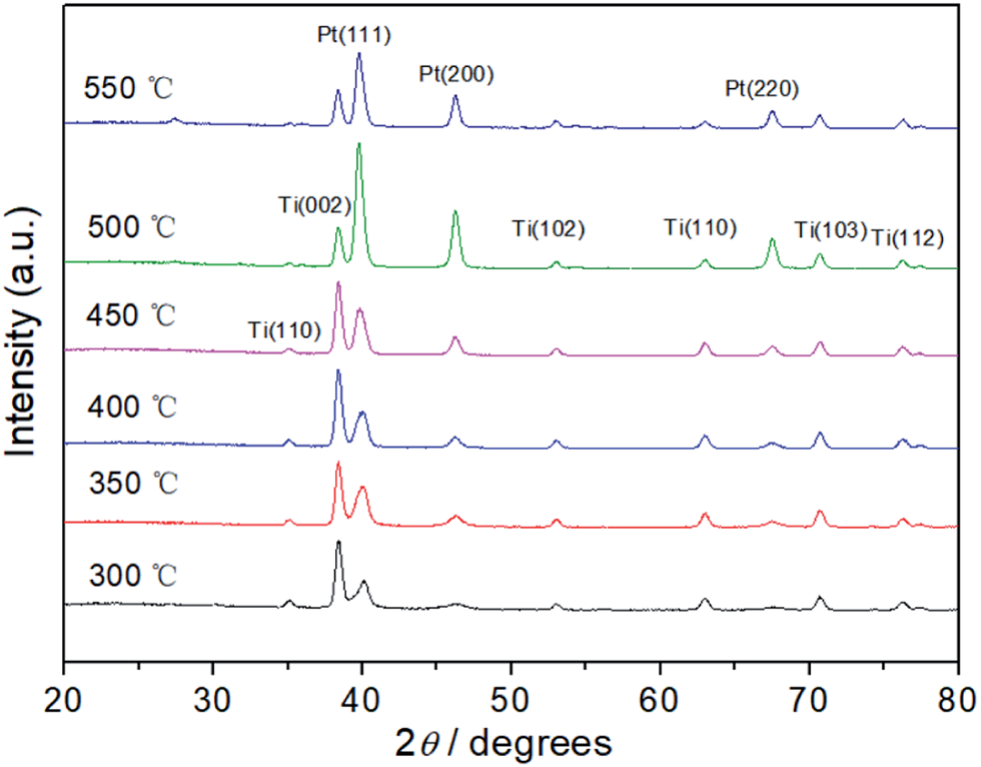
X-ray diffraction curves of Pt electrodes with different thermal decomposition temperatures.

The crystal structures of Pt electrodes with different thermal decomposition temperatures were characterized by XRD (Fig. 2). The characteristic diffraction peaks of (111), (200), and (220) crystal facets are observed clearly, indicating that the Pt electrodes possess the face-centered cubic (fcc) crystal structure compared with JCPDS no. 04-0802. In addition, diffraction peaks of Ti matrix can also be observed according to JCPDS no. 44-1294, which indicate that the surface thickness of Pt film is relatively thin. Further, the grain size of the Pt particle was calculated according to the peak width of well-shaped (200) reflection. In Fig. S1,† with the increase of thermal decomposition temperature, the particle size has increased from 5.8 nm (300 °C) to 16.2 nm (550 °C) due to high temperature sintering.

**Fig. 3:**
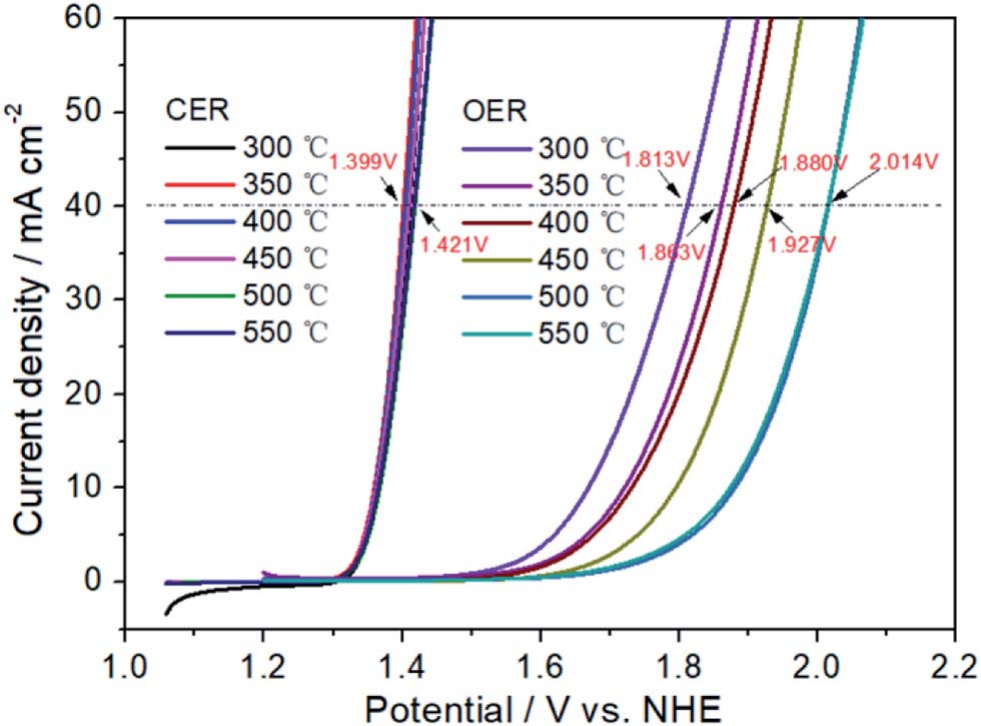
CER and OER activities of Pt electrodes with different thermal decomposition temperatures.

**Fig. 4:**
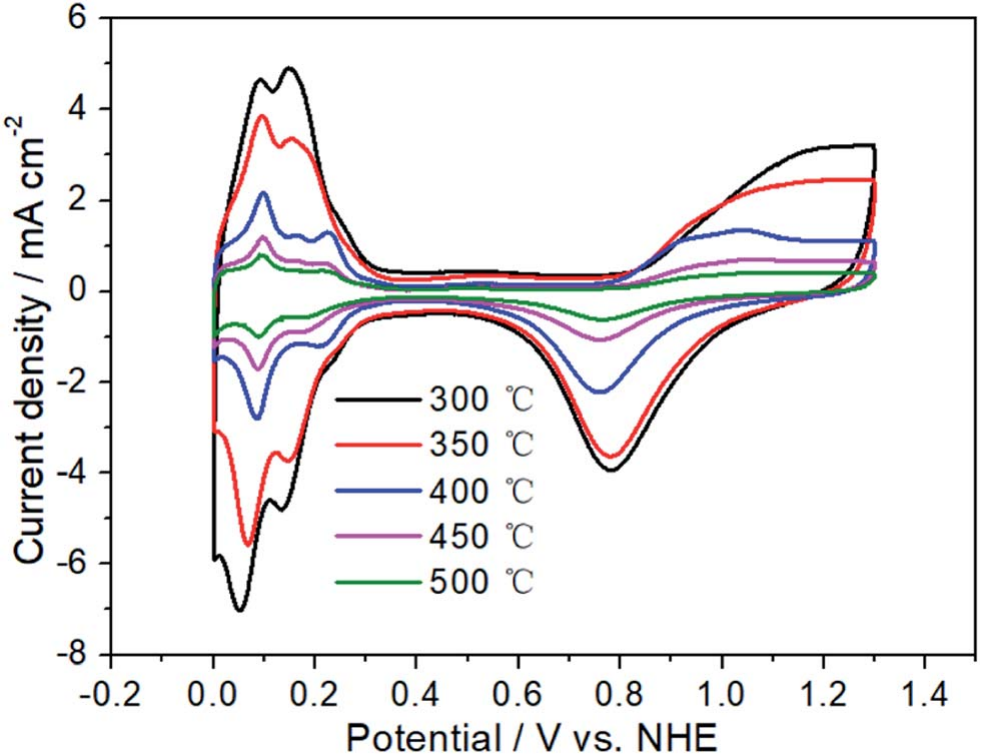
Cyclic voltammograms of Pt electrodes with different thermal decomposition temperatures.

Fig. 3 has exhibited the chlorine evolution reaction (CER) and oxygen evolution reaction (OER) activities of Pt electrodes with different thermal decomposition temperatures. First of all, it can be seen that their CER activities are very close. When the current is 40 mA cm^−2^, the potential range of CER is 1.399–1.421 V with the variation of only 22 mV. However, for OER activity, the potential variation is 201 mV (from 1.813 to 2.014 V), which is 10 times that of CER. When the thermal decomposition temperature is 300 °C, the OER potential is 1.813 V to obtain the current of 40 mA cm^−2^. However, for Pt-350, Pt-400 and Pt-450, the OER potential has been increased to 1.863, 1.880 and 1.927 V, respectively. Especially for Pt-500 and Pt-550, the OER potential is 2.014 V. At this time, the potential difference between CER and OER (Δ*E*_CER–OER_) has increased to 597 mV, while the Δ*E*_CER–OER_ of Pt-300 electrode is only 401 mV. A high Δ*E*_CER–OER_ would facilitate the CER and inhibit the OER.^58,59^ Therefore, the increasement of thermal decomposition temperature, should enhance the selectivity of CER. In addition, the sharp decrease of OER activity may be related to the variation of the electrochemical surface area (ESA). In the range of 300–500 °C, the higher of thermal decomposition temperature, the smaller the oxidation–reduction current of Pt electrode surface in Fig. 4. For Pt-500 and Pt-550, their currents are basically the same (Fig. S2†). Further calculations have shown that when the temperature rises from 300 to 500 °C, the ESA has dropped from 90 to 11 cm^2^ (Fig. S3†), reducing by 9 times. However, the reduction of ESA has little effect on CER activity. Therefore, it provides the possibility to change the CER selectivity of the platinum electrode by adjusting the calcination temperature.

**Fig. 5:**
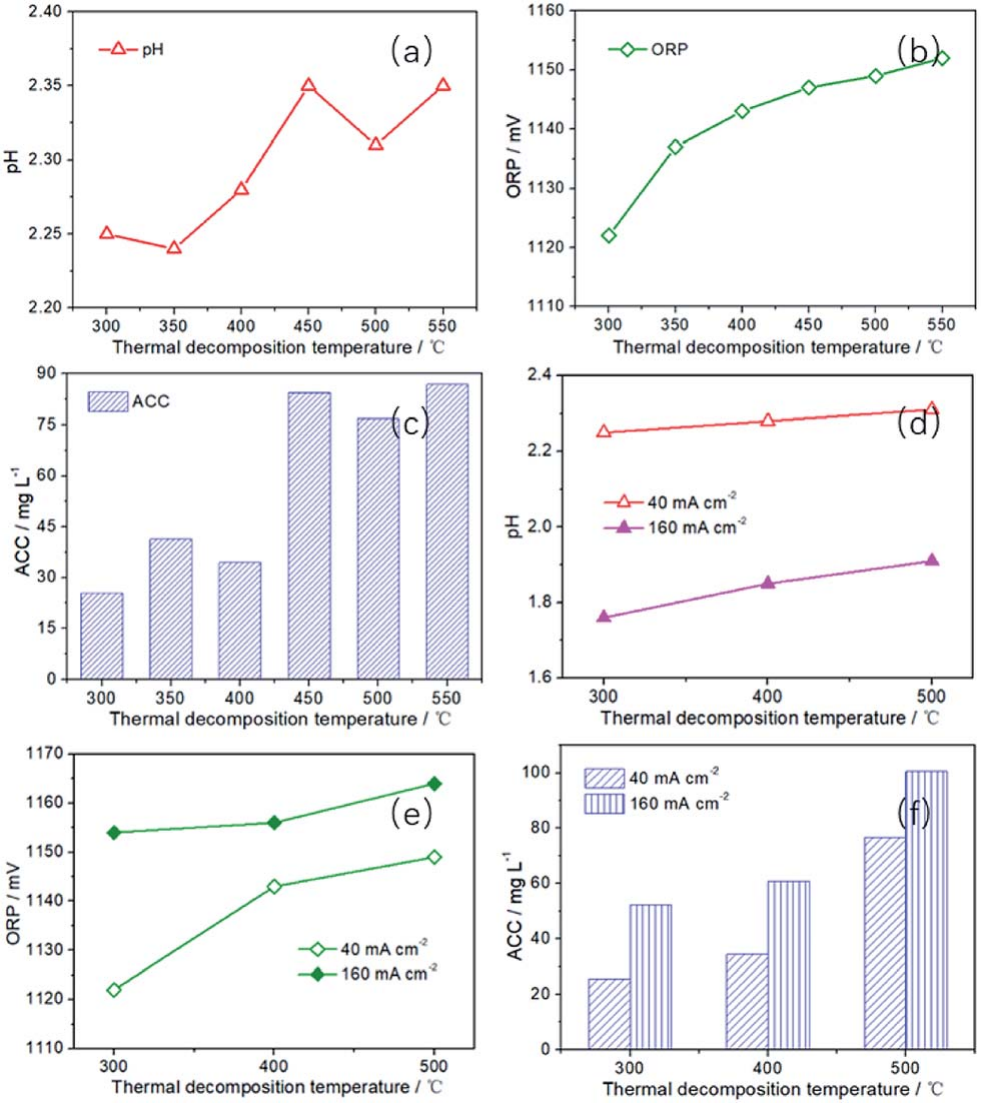
The pH, ORP and ACC of AEOW prepared by Pt electrodes with different thermal decomposition temperatures (preparation conditions: *I* = 40 and 160 mA cm^−2^, *t* = 30 min, *C*_NaCl_ = 0.5 g L^−1^).

Since SAEW was obtained by mixing cathode solution (AERW) and anode solution (AEOW). In order to gain SAEW efficiently, the performance of AEOW prepared by a series of platinum electrodes with different thermal decomposition temperatures was first studied. With the increase of thermal decomposition temperature, the pH, ORP and ACC values all show an upward trend in Fig. 5a–c. It is because Pt electrode with high decomposition temperature has high CER selectivity. High CER selectivity can inhibit the OER, thus reducing the [H^+^] concentration in the anode region (increasing pH value). Moreover, the improvement of CER selectivity is conducive to enhance the [Cl_2_] content in the solution, thus increasing the ACC and ORP (*E*(Cl_2_/Cl^−^)) values. The ACC is 25.48, 41.35 and 34.42 mg L^−1^, respectively, corresponding to the Pt-300, Pt-350 and Pt-400. When the thermal decomposition temperature is raised to 450–550 °C, the ACC is 76.77–84.48 mg L^−1^, which is 3.01–3.32 times that of Pt-300. For the ORP value, the value has risen from 1122 to 1152 mV with thermal decomposition temperature ranging from 300 to 550 °C. In addition, when the current density is increased to 160 mA cm^−2^, the pH value decreases sharply (40 mA cm^−2^: 2.24–2.35; 160 mA cm^−2^: 1.76–1.91), while the ORP value (40 mA cm^−2^: 1122–1149 mV; 160 mA cm^−2^: 1154–1164 mV) and ACC value (40 mA cm^−2^: 25.48–84.48 mg L^−1^; 160 mA cm^−2^: 52.30–100.58 mg L^−1^) increases significantly in Fig. 5d–f. However, these characteristic values all continue to rise with the increase of thermal decomposition temperature. The pH value has increased from 1.76 to 1.91, while the ORP value has increased from 1154 to 1164 mV, and the ACC value has increased from 52.30 to 100.58 mg L^−1^. Therefore, for AEOW, Pt-450, Pt-500 and Pt-550 electrodes are very suitable, while other electrodes are less efficient in producing available chlorine.

**Fig. 6:**
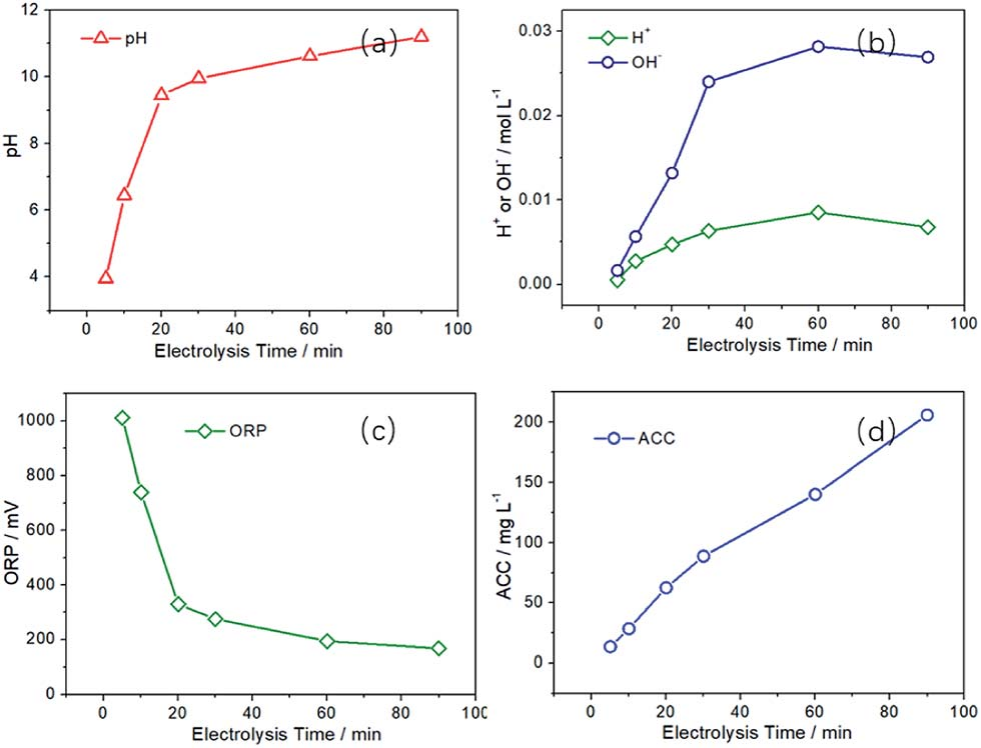
Effect of electrolysis time on the pH, ORP and ACC values of SAEW and the content of H^+^ and OH^−^ in anode and cathode region. (*I* = 60 mA cm^−2^, *C*_NaCl_ = 0.5 g L^−1^).

Then the Pt-500 electrode was used in the following investigation of SAEW preparation process. First, the effect of electrolysis time on the preparation of SAEW were discussed. As can be seen from Fig. 6a, with the extension of electrolysis time, the pH value of SAEW first increases rapidly and then gradually becomes stable. When the electrolysis time is 5 min, the pH is 3.95, which is obviously acidic. Then the pH becomes 6.45 at electrolysis time 10 min, which is slightly acidic. However, as the electrolysis time is increased to 20 min, the pH is 9.45, showing an alkaline characteristic. In addition, when the electrolysis time is 5–10 min, the ORP of SAEW is relatively high (740–1011 mV) in Fig. 6c. When the electrolysis time exceeds 10 min, the ORP decreases drastically to 329 mV (20 min) and 167 mV (90 min), respectively.

This is because the following three reactions mainly take place in the cathode and anode during the electrolysis process.^58^Cathode: 2H_2_O + 2e = H_2_ + 2OH^−^, −0.8277 VAnode: 2Cl^−^ − 2e = Cl_2_, 1.35827 V2H_2_O − 4e = O_2_ + 4H^+^, 1.229 V

During the preparation process, water molecules in the cathode region can gain electrons from the electrode to generate hydrogen (hydrogen evolution reaction, HER), and it will directly increase [OH^−^] content (increase the pH value) in the solution. However, there are two competing reactions of CER and OER in the anode region. The occurrence of OER will directly produce [H^+^] and cause the solution to become acidic. While the CER can generate chlorine and hypochlorous acid and make the solution contain a certain amount of ACC. Since there is only one main reaction in the cathode region and two competing reactions in the anode region, the concentration of [OH^−^] in the cathode region is higher than that of [H^+^] in the anode region (Fig. 6b). Moreover, with the extension of electrolysis time, the concentration difference between [H^+^] and [OH^−^] will gradually increase. Therefore, the solution is alkaline after mixing the anode and cathode region solution.

The ORP depends on the value of *E*(H_2_O/O_2_, H^+^), *E*(H_2_O/H_2_, OH^−^) and *E*(Cl_2_/Cl^−^), where the values of *E*(H_2_O/O_2_, H^+^) and *E*(H_2_O/H_2_, OH^−^) would decrease with the reduce of [H^+^] in solution. Therefore, as the electrolysis time is prolonged, the ORP value will decrease significantly in Fig. 6c. In addition, the following reaction will occur (Cl_2_ + 2NaOH = NaCl + NaClO + H_2_O) in the cathode and anode mixed solution, reducing the concentration of [Cl_2_]. The decrease of [Cl_2_] will reduce the ORP value, although *E*(Cl_2_/Cl^−^) is not affected by the pH value of solution. Fig. 6d is the effect of electrolysis time on the ACC. The ACC is 28.40 mg L^−1^ at 10 min, while the ACC is 140.23 mg L^−1^ at 60 min, with a five-fold increase.

**Fig. 7:**
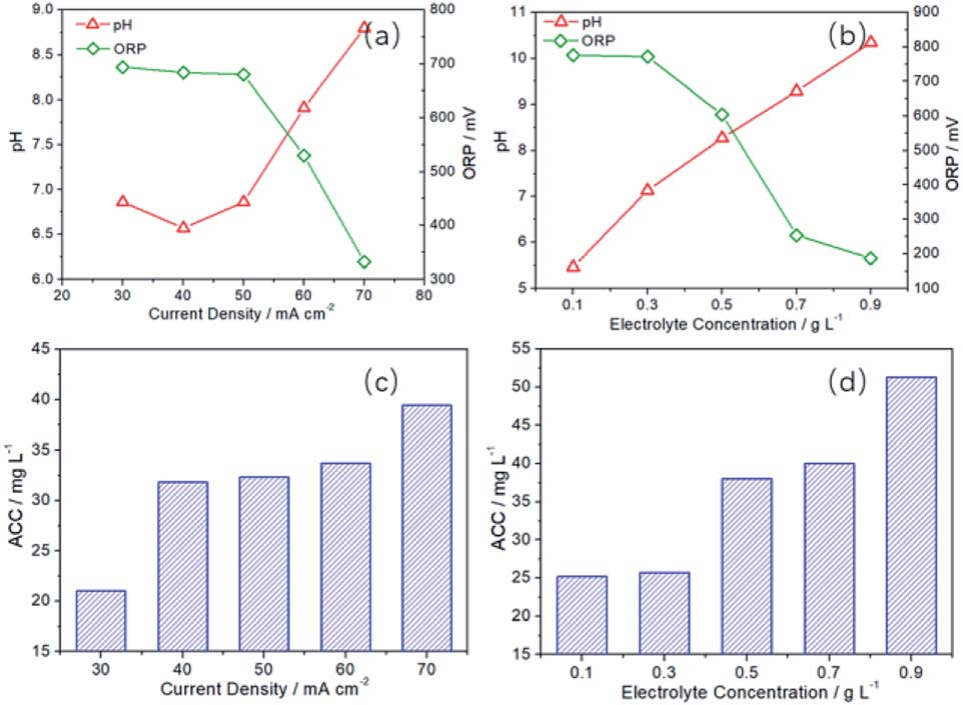
Effect of current density (*t* = 10 min, *C*_NaCl_ = 0.5 g L^−1^) and electrolyte concentration (*t* = 20 min, *I* = 60 mA cm^−2^) on the pH, ORP and ACC values of SAEW.

Then, the effects of current density and electrolyte concentration on the preparation of SAEW were further investigated. Obviously, the increase of electrolyte concentration (NaCl) and current density could be conducive to promote the electrochemical reaction rate in the anode and cathode region. Moreover, it is well known that the increase of NaCl concentration would facilitate the CER reaction in the anode.^60,61^ Meanwhile, as can be seen from Fig. S4,† Δ*E*_CER–OER_ has been increased from 581 to 632 mV as the current density increases from 30 to 80 mA cm^−2^. Therefore, increasing electrolyte concentration^62–65^ and current density^66–68^ is beneficial to improve the CER selectivity and decrease the OER selectivity. With the increasing of current density and electrolyte concentration, the current efficiency of OER (generating H^+^) in the anode region will be suppressed. In the cathode region, the increase of current density and electrolyte concentration will not affect the current efficiency of HER (generating OH^−^), which remains unchanged. Therefore, the pH value of SAEW obtained by mixing the solution in the anode and cathode region will increase. In Fig. 7a, when the current density is 30–50 mA cm^−2^, the pH value is relatively low (6.57–6.86). However, the pH value has been increased to 7.91 (60 mA cm^−2^) and 8.80 (70 mA cm^−2^), respectively. Contrary to the change trend of pH value, ORP value has shown a downward trend. The ORP value is reduced from an initial 694 mV (30 mA cm^−2^) to 530 mV (60 mA cm^−2^) and then further down to 332 mV (70 mA cm^−2^).

The effect of electrolyte concentration on the pH and ORP values is similar to that of the current density. They both make the pH and ORP of the solution change by adjusting the CER selectivity. The pH is 5.46 at the electrolyte concentration 0.1 g L^−1^, which is slightly acidic. When the electrolyte concentration is 0.3–0.5 g L^−1^, the pH is 7.13–8.27, which close to neutral at this time. But as the electrolyte concentration is 0.7–0.9 g L^−1^, the pH is 9.29–10.35, which is slightly alkaline. The ORP is 772–776 mV at the electrolyte concentration 0.1–0.3 g L^−1^, which has relatively high redox potential. When the electrolyte concentration is 0.7–0.9 g L^−1^, the ORP decrease rapidly to 254 mV and 186 mV.

In addition, it can be seen from Fig. 7c and d that the ACC increases obviously with the increasement of the current density and electrolyte concentration. When the current density is 30 mA cm^−2^, the ACC was 21.03 mg L^−1^. When the current density is 70 mA cm^−2^, ACC is 39.41 mg L^−1^, which is almost twice as high as that of 30 mA cm^−2^. The production of available chlorine is dependent on the CER activity. Therefore, the enhancement of current density can accelerate the rate of CER, thus increasing the ACC content. When the electrolyte concentration is changed from 0.1 g L^−1^ to 0.9 g L^−1^, the ACC rises from 25.21 mg L^−1^ to 51.30 mg L^−1^, which almost doubled. This is because the increase of electrolyte concentration is beneficial to reduce the concentration polarization of CER in the anodic region, which increase the mass transfer driving force.^69,70^ Thus, the more chlorine gas, hypochlorous acid, chlorine dioxide and other high-priced chlorine compounds is produced.^71–73^

## Conclusions

First, the effects of Pt electrodes with different thermal decomposition temperatures on AEOW's pH, ORP and ACC values were systematically investigated in this paper. With the increase of thermal decomposition temperature, the pH, ORP and ACC value all have shown an upward trend. It is because Pt electrode with high decomposition temperature has a good CER selectivity. Furthermore, the CER and OER activity of platinum electrodes were characterized by linear voltammetry scanning. For Pt-500 and Pt-550, the Δ*E*_CER–OER_ has reached 597 mV, while the Δ*E*_CER–OER_ of Pt-300 electrode was only 401 mV. A high potential difference of Δ*E*_CER–OER_ would facilitate the chlorine evolution reaction and inhibit the oxygen evolution reaction. Next, the electrolysis process for SAEW preparation was studied systematically. The process is based on the preparation of AEOW by ion-exchange membrane electrolysis, reasonably mixing the electrolyzed cathode and anode solution to obtain SAEW. The results showed that with the increase of electrolysis time, current density and electrolyte concentration, the SAEW's pH value increased, ORP decreased and ACC increased. Through the investigation of above process conditions, SAEW can be efficiently prepared at 0.1–0.3 g L^−1^ NaCl, 30–50 mA cm^−2^ and 10 min.

## Conflicts of interest

There are no conflicts to declare.

## Supplementary Material

RA-009-C8RA08929A-s001
