# Supplementary material for: Effect of platinum electrode materials and electrolysis processes on the preparation of acidic electrolyzed oxidizing water and slightly acidic electrolyzed water
Source: RSC Adv. 2019 Jan 22;9(6):3113–9. doi: 10.1039/c8ra08929a (PMC9059949; doi:10.1039/c8ra08929a)
Supplement: RA-009-C8RA08929A-s001 [file RA-009-C8RA08929A-s001.pdf]

Electronic Supplementary Material (ESI) for RSC Advances.
This journal is © The Royal Society of Chemistry 2019

Electronic Supplementary Information

Effect of platinum electrode material and electrolysis process on the preparation of acidic electrolyzed oxidizing water and slightly acidic electrolyzed water

Xiang Song, Hui Zhao, Keneng Fang, Yongshan Lou, Zongkui Liu, Chifeng Liu, Zhandong Ren,* Xiaorong Zhou, Hua Fang and Yuchan Zhu*

School of Chemical and Environmental Engineering, Wuhan Polytechnic University, Wuhan, 430023, P. R. China.

E-mail: renzhandong@163.com, zhuyuchan@163.com

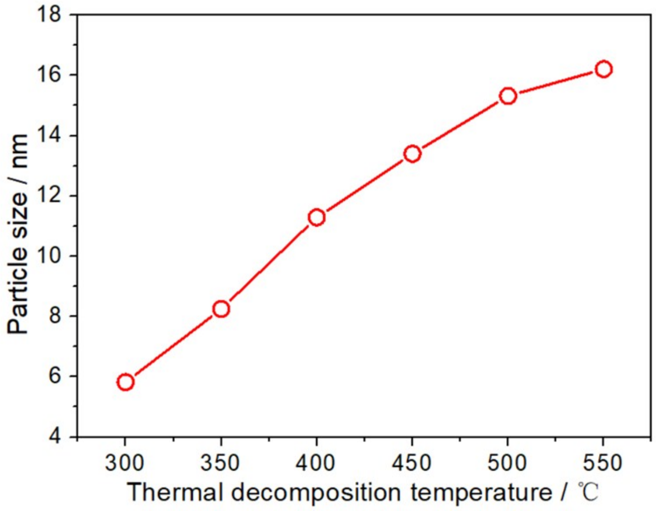

Figure S1 Particle size of Pt electrodes with different thermal decomposition temperatures.

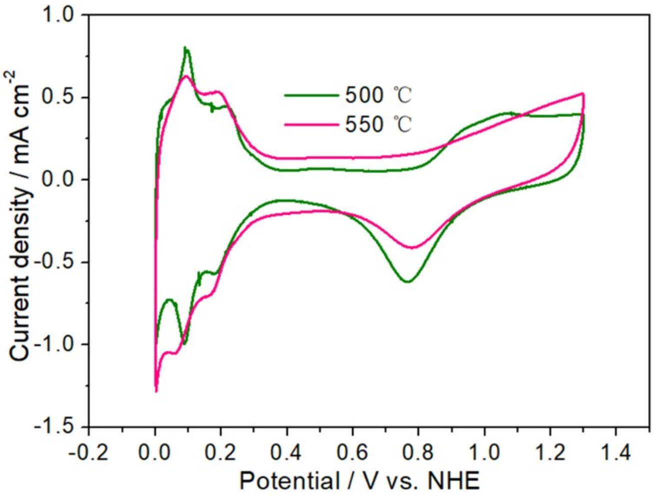

Figure S2 Cyclic voltammogram for Pt electrodes with thermal decomposition temperature 500 ℃ and 550 ℃.

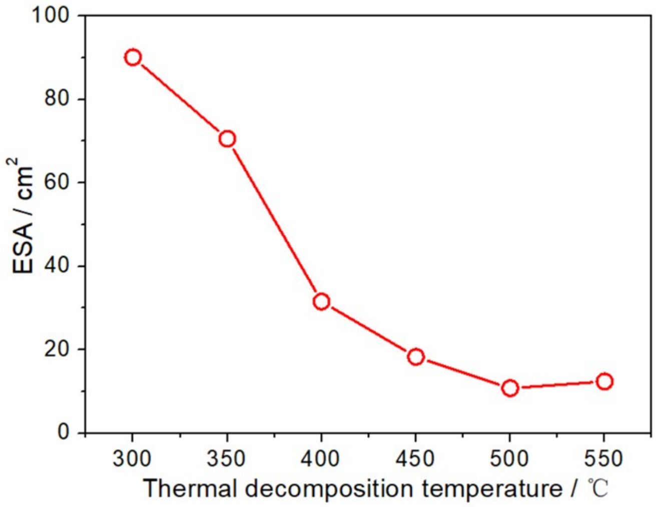

Figure S3 Electrochemical surface area (ESA) of Pt electrodes with different thermal decomposition temperatures.

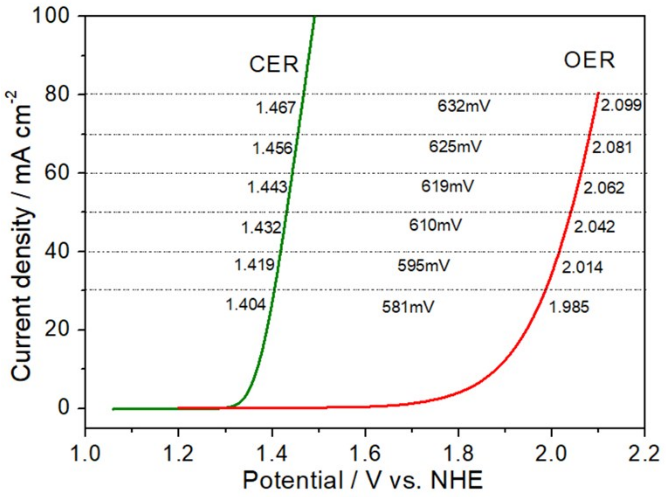

Figure S4 The potential difference between CER and OER ($\Delta E_{CER-OER}$) with different current density
